# Severe Acute Respiratory Syndrome: Clinical Outcome and Prognostic Correlates^1^

**DOI:** 10.3201/eid0909.030362

**Published:** 2003-09

**Authors:** Ping Tim Tsui, Man Leung Kwok, Hon Yuen, Sik To Lai

**Affiliations:** * Princess Margaret Hospital, Hong Kong, China

**Keywords:** severe acute respiratory syndrome, clinical outcome, ribavirin, steroid

## Abstract

Severe acute respiratory syndrome (SARS) poses a major threat to the health of people worldwide. We performed a retrospective case series analysis to assess clinical outcome and identify pretreatment prognostic correlates of SARS, managed under a standardized treatment protocol. We studied 127 male and 196 female patients with a mean age of 41±14 (range 18–83). All patients, except two, received ribavirin and steroid combination therapy. In 115 (36%) patients, the course of disease was limited. Pneumonitis progressed rapidly in the remaining patients. Sixty-seven (21%) patients required intensive care, and 42 (13%) required ventilator support. Advanced age, high admission neutrophil count, and high initial lactate dehydrogenase level were independent correlates of an adverse clinical outcome. SARS-associated coronavirus caused severe illnesses in most patients, despite early treatment with ribavirin and steroid. This study has identified three independent pretreatment prognostic correlates.

The outbreak of severe acute respiratory syndrome (SARS) in Hong Kong was caused by a novel virus belonging to the family *Coronaviridae* (*1*,*2*). The virus is transmitted through respiratory droplets, direct contact with fomites, and aerosolized respiratory secretions (*3*,*4*). The first outbreak was linked to an index patient treated in the Prince of Wales Hospital (*4*). The second wave of spread in the community was started by an infected patient with renal disease and amplified by the sewage system of Amoy Gardens, a densely populated condominium in Hong Kong (*5*). The floor drain traps in many apartments of Amoy Gardens were not filled with water and thus lost the sealing function. Therefore, the bathrooms of many apartments were openly connected with the soil stack. Virus-loaded droplets of an affected apartment could have been spread through the floor drain system. Hundreds of patients were then treated in public hospitals. The virus was highly contagious and caused substantial illness and death among the general population as well as among healthcare workers.

The Hong Kong Hospital Authority, which provides more than 90% of inpatient care in Hong Kong, has been responsible for the management of all SARS patients (*6*). The Princess Margaret Hospital is a designated treatment center for SARS patients. Convalescent-phase SARS patients are treated in Wong Tai Sin Hospital. More than 500 SARS patients have been treated in these two hospitals since March 2003. The Hospital Authority has established a structured approach in the diagnosis, investigation, and treatment of SARS. The clinical diagnostic criteria of the Hospital Authority’s SARS registry (defined in Table 1) were similar to the case definition of probable SARS by the World Health Organization (*3*).

**Table 1:** Case definition of SARS, Hong Kong Hospital Authority SARS Registry, April 22, 2003^a,b^




Inclusion criteria	Exclusion criterion

Radiographic evidence of infiltrates consistent with pneumonia	A case should be excluded if an alternative diagnosis can fully explain the illness
Temperature >38°C or history of such temperature at any time in the past 2 days	
At least two of the following:	
History of chills in the past 2 days Cough (new or increased cough) or breathing difficulty General malaise or myalgia Known history of exposure	

^a^SARS, severe acute respiratory syndrome.

Persons infected with the SARS-associated coronavirus may exhibit a wide spectrum of signs and symptoms and a varied clinical course. We have found asymptomatic cases and patients with spontaneous recovery without antiviral or steroid therapy (*7*); SARS is at the other end of the disease spectrum. The Hospital Authority’s hypothetical disease model has three phases: viral replication, immune hyperactivity, and pulmonary destruction (*8*). Autopsy findings have supported the theory of cytokine deregulation in SARS (*9*). Using steroids in the treatment of SARS was based on this hypothesis and on initial clinical experience in the management of SARS in Hong Kong (*4*).

The recommended treatment regime at the time of the Amoy Gardens outbreak consisted of antibiotics, ribavirin, and steroid combination therapy. Patients without known epidemiologic contact with SARS patients were treated with antibiotics that would prevent both community- acquired pneumonia and hospital infections. If patients did not respond to antibiotics in 48 h, they would be given a combination of ribavirin and steroid. For patients with an epidemiologic history of contact with a SARS patient, this combination would be started together with the above antibiotic. Ribavirin would be given at a dose of 8 mg/kg intravenously every 8 h. For patients who appeared for treatment with extensive pneumonitis, a loading dose of 33 mg/kg of ribavirin, followed by 20 mg/kg every 8 h, was given intravenously. Hydrocortisone, 2 mg/kg every 6 h or 4 mg/kg every 8 h, would be administered, together with ribavirin. Oral equivalent doses of ribavirin and prednisolone could be prescribed at any stage of the disease. The total duration of therapy could range from 14 to 21 days. Besides administering steroids, we have tried in selected cases immunomodulation through the use of intravenous pentaglobin. Pulsed doses of methylprednisolone were restricted to those with disease progression and marked lung involvement. Lee et al. have made a comprehensive report of 138 cases of suspected SARS during a hospital outbreak in Hong Kong (*4*). Our study investigated the SARS patients after the Amoy Gardens outbreak to identify associated pretreatment prognostic factors for risk stratification and assess the clinical outcome of SARS under a standardized treatment protocol.

## Methods

We performed a retrospective case series study. All reported SARS patients who stayed in the medical wards or intensive care unit of Princess Margaret Hospital and Wong Tai Sin Hospital on April 16, 2003, were screened. Patients were excluded if subsequent follow-up serologic tests showed no rise in antibody titer against SARS-associated coronavirus. All eligible SARS patients, except three, were recruited into the study. One healthcare worker refused to be studied, and two patients who were suspected of contracting the infection during their hospital stay were also excluded. This cohort was followed up until May 20, 2003. Data were collected through the hospital authority’s computerized clinical management system, case record review, and a questionnaire survey assisted by the nursing staff of each SARS ward. Age, sex, occupation, residential address, smoking habit, time between onset of fever and start of antiviral therapy, coexisting conditions, and laboratory data were the variables under study. Outcome variables were the following: dependency on high amounts of oxygen (requiring at least 3 L/min of oxygen through a nasal cannula) and admission to an intensive care unit or death.

### Statistical Analysis

Categorical variables were analyzed with the chi-square test and the means of continuous variables were compared with the Student t test. Association among continuous variables was assessed with Pearson correlation coefficient. Multivariate logistic regression by backward stepwise analysis was performed to identify independent variables that correlated with the clinical outcome as of May 20, 2003. Cox’s regression model was used to study survival data. Plus-minus values are mean ± standard deviation; a p value of <0.05 was considered significant, and all probabilities were two-tailed. SYSTAT software (version 10.0, SPSS, Chicago, IL) was used for statistical analysis.

## Results

The study population consisted of 127 male and 196 female patients, ranging in age from 18 to 83 (41±14). Forty-seven (15%) patients were healthcare workers. One hundred thirty-three (41%) were Amoy Gardens residents. Two hundred seventy-three (85%) patients were in good health. The coexisting conditions are listed in Table 2. Psychiatric illness, hepatitis B carrier status, and thalassemia trait status were not classified as coexisting conditions. Fifteen (14%) males and 7 (4%) females were current smokers. The overall prevalence of smoking among SARS patients was 7.6% (9.1% if healthcare workers are excluded). None of the affected healthcare workers smoked. The symptoms exhibited fulfilled the diagnostic criteria of the Hospital Authority’s SARS registry.

**Table 2:** Coexisting conditions in patients with severe acute respiratory syndrome

Coexisting condition	No. of patients
Hypertension	16
Diabetes mellitus	8
Chronic lung disease	6
Pregnancy	5
Neurologic disease	5
Renal disease	4
Cardiovascular disease	3
Immunologic disease	3
Malignancy	1

All patients had lung involvement, documented either by chest x-ray or high-resolution computed tomographic scan of the thorax. Lymphopenia, found in 221 (68%) patients, was a prominent feature in those who sought treatment. Other initial laboratory findings included thrombocytopenia (41%), elevated creatine kinase level (14%), and elevated lactate dehydrogenase level (42%). Initial bacterial cultures were negative. Virus screening was negative for adenovirus, respiratory syncytial virus, influenza A and B, and parainfluenza virus. Two hundred and seven (64%) patients had reverse transcriptase–polymerase chain reaction (RT-PCR) assays performed for SARS-associated coronavirus, and 128 (62%) of the results were positive. Two hundred and forty-two (75%) patients had completed serologic testing. The diagnosis of recent SARS-associated coronavirus infection was confirmed by either RT-PCR assays or serologic test in 286 (89%) patients. The sensitivity of RT-PCR assays was 58% (95% confidence interval [CI], 50% to 66%).

Our patients sought treatment 3.9±2.7 days after onset of fever. The interval between onset of fever and admission was positively correlated with admission neutrophil count (Pearson r=0.1, p=0.07), admission platelet count (Pearson r=0.1, p=0.06), and initial lactate dehydrogenase level (Pearson r=0.36, p<0.001). An antibiotic was started immediately after admission in all cases. Either levofloxacin, 500 mg once a day, or amoxicillin/clavulinate acid, 375 mg three times a day plus clarithromycin, 500 mg twice a day, was used to protect against community-acquired pneumonia. All patients were also treated with oral or intravenous ribavirin, according to protocol. Most (94%) were given either intravenous hydrocortisone or oral prednisolone, according to protocol. Five patients received intravenous methylprednisolone as a form of steroid therapy. The dose was administered at 3 mg/kg once a day and would be tapered down to 1 mg/kg if the patient showed a clinical response. Pulsed doses of methylprednisolone (500 mg per dose) were given as initial treatment in 12 patients, who then received maintenance steroid therapy. Two patients were treated with ribavirin only. Ribavirin plus steroid therapy was administered 1.2±1.7 days after admission. The interval between admission and initiation of antiviral therapy was negatively correlated with the interval between onset of fever and admission (Pearson r = –0.17, p=0.003).

### Clinical Outcome

In 115 (36%) patients, the disease was limited with resolution of fever and pneumonitis. Two hundred and eight (64%) patients had either clinical or radiologic evidence of progression of pneumonitis, and they received 2.9±2 gm pulsed dose methylprednisolone therapy. Maintenance steroid was resumed after pulsed dose therapy. Patients who were given pulsed doses of steroids were treated with potent broad-spectrum intravenous antibiotics (piperacillin and tazobactam) to protect against hospital-acquired infection. Hyperglycemia, hypokalemia, flare up of hepatitis B infection, hospital-acquired infection, and steroid psychosis were the acute side effects encountered. Hepatitis B carriers were treated with lamivudine, 100 mg once a day; no liver failure occurred in members of this cohort. Disease progression was apparently arrested by pulsed dose steroid therapy in 98 (30%) patients. In the remaining 110 (34%) patients, the illness ran a severe and protracted course, and the patient needed high doses of oxygen. Sixty-seven (21%) had been admitted to intensive care unit, and 42 (13%) required ventilator support. Twenty-six patients died (12 males and 14 females). The crude mortality rate of our cohort after 47±8 days of follow-up was 7.9% (95% CI, 5% to 10.8%) and was an underestimation because of sampling bias. Those who died before April 16, 2003, were excluded from our sample, while long-term survivors were retained for study. Among them, 10 had concurrent medical illness. No healthcare worker in this cohort died. Diabetes was found in three patients who died, and hypertension in four who died. Eleven of those who died lived in Amoy Gardens. A young pregnant woman died after delivery, despite aggressive treatment.

**Figure 1:**
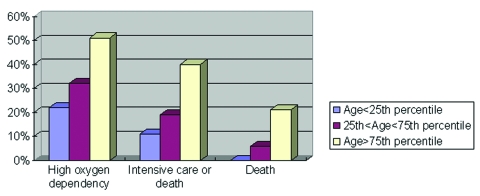
Relationship between age and fatal severe acute respiratory syndrome illness, Hong Kong, 2003.

**Figure 2:**
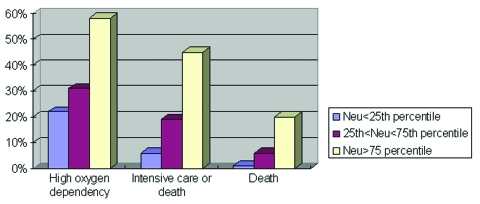
Relationship between neutrophil count and fatal severe acute respiratory syndrome illness, Hong Kong, 2003.

**Figure 3:**
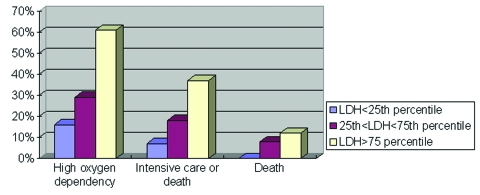
Relationship between lactate dehydrogenase level (LDH) and fatal severe acute respiratory syndrome illness, Hong Kong, 2003

Age, sex, healthcare worker status, Amoy Gardens resident status, presence of coexisting conditions, interval between onset of fever and therapy (ribavirin plus steroid), neutrophil and platelet count on admission, and initial creatine kinase and lactate dehydrogenase levels were the correlates of clinical outcome under study. Variables with a p value of <0.1 by univariate analysis were entered into the multivariate regression model. By multivariate logistic regression, advanced age, high neutrophil count on admission, and high initial lactate dehydrogenase level were independent correlates of high oxygen dependency as well as intensive care unit admission or death (Table 3). By Cox’s backward stepwise regression, young age, low neutrophil count on admission, and healthcare worker status (p=0.05) were favorable independent correlates of survival time (Table 3). A dose-response relationship also existed between the independent correlates and clinical outcome (Figure 1,2,3). We used the term “correlates” instead of “predictors” of outcome because of the method we used, a case series.

**Table 3:** Independent prognostic correlates and clinical outcome

Correlates	High oxygen dependency	ICU care or death	Survival time
	OR (95% CI) p value	OR (95% CI) p value	Hazard ratio (95% CI) p value
Age (per 10-y increase)	1.48 (1.21 to 1.8) p<0.001	1.57 (1.26 to 1.95) p<0.001	1.75 (1.38 to 2.2) p<0.001
Admission neutrophil (per 1x10^9^/L increase)	1.31 (1.14 to 1.5) p<0.001	1.28 (1.13 to 1.46) p<0.001	1.17 (1.09 to 1.26) p<0.001
Initial LDH level (per 100 IU/L increase)	1.49 (1.23 to 1.82) p<0.001	1.35 (1.11 to 1.64) p=0.003	p value not significant

^a^ICU, intensive care unit; LDH = lactate dehydrogenase; OR, odds ratio; CI, confidence interval.

The second serology titer obtained after the end of second week was negatively correlated with age (Pearson r=–0.13, p=0.05) and admission lymphocyte count (Pearson r=–0.17, p=0.01). Conversely, the neutrophil count on admission was positively correlated with the second serology titer (Pearson r=0.2, p=0.003). The pulsed dose of steroid was not shown to affect the second serology titer (Pearson r=0.1, p=0.18). Patients who depended on high oxygen therapy had a higher second antibody titer against SARS-associated coronavirus (p = 0.05).

## Discussion

The virus attacked persons of both sexes and all ages. Many were previously in good health and the wage earners in their families. Not infrequently, several members of a family were admitted to the hospital. The need for isolation discouraged close social contact. Unfortunately, some of the patients were also stigmatized. The psychosocial effect of SARS is by no means a lesser problem.

RT-PCR assay for SARS-associated coronavirus is a new test, and its sensitivity and specificity have yet to be established. In our cohort, the sensitivity was 58%, and results depended on sampling technique and stage of disease (*10*). Contamination of specimen could lead to a false-positive result. A false-negative result could arise from performing the test in the very early or late stage of the disease. Diarrhea was common among Amoy Gardens SARS patients. The virus could be found in stool by RT-PCR assays. A negative test does not rule out the diagnosis, however. The serologic test remains as the standard criterion of definitive diagnosis. Pulsed doses of steroid did not seem to affect the humoral response of SARS patients. In retrospect, the intensity of antibody response was related to clinical outcome and associated pretreatment prognostic factors. The viral load could be a determinant of these prognostic association factors.

Our hematologic and biochemical data, as well as associated prognostic factors, agreed with the work of Lee et al. (*4*). Both advanced age and high neutrophil count on admission were associated with poor outcome. We found that initial lactate dehydrogenase level was also an associated prognostic factor. The early phase of SARS is characterized by lymphopenia and thrombocytopenia. As the disease progresses, both neutrophil and platelet counts rise, accompanied by an elevation in lactate dehydrogenase level. The neutrophilic response is important in the pathogenesis of hypersensitivity pneumonitis, and thus the initial neutrophil count could also indicate disease progression. Lactate dehydrogenase level reflects tissue necrosis related to immune hyperactivity in SARS and thus relates to poor outcome. Patients with high neutrophil counts and lactate dehydrogenase levels on admission could have been late in seeking treatment or have experienced heavy exposure to the virus.

### Effect on Healthcare Workers

The spread of the disease to healthcare workers is a major problem in any country dealing with SARS. Intubation, nasopharyngeal aspiration, chest physiotherapy, handling of excreta, and even feeding become high-risk procedures. All healthcare workers working in Hospital Authority hospitals are required to follow the recommended personal protection equipment standards (*11*). The level of precaution depends on the risk in the work area and the type of procedure performed. All healthcare workers working in a SARS area wore N-95 masks, face shields, caps, gowns, and surgical gloves. The intensive care unit was high-risk area in this cohort (Table 4). However, healthcare workers working in a non-SARS area were not exempted. They contracted the disease from SARS patients who sought treatment early or exhibited atypical signs and symptoms. By univariate analysis, healthcare worker status was negatively correlated with death. Healthcare workers were younger. They sought treatment earlier and had a lower neutrophil count and lower initial lactate dehydrogenase level on admission (Table 5). Nevertheless, healthcare worker status was still an independent survival correlate after controlling these confounding variables. The current safety precaution could not prevent all frontline healthcare workers from contracting SARS, but minimizing individual exposure to the virus might reduce the viral load, subsequent immune hyperactivity, and the risk for a fatal outcome.

**Table 4:** Number of infected healthcare workers treated in Princess Margaret Hospital, Hong Kong, 2003^a^

	ICU	SARS area	Non-SARS area	Total no. (%)
Doctor	2	1	1	4 (9)
Nurse	9	15	3	27 (57)
Other	6	3	7	16 (34)

^a^ICU, intensive care unit; SARS, severe acute respiratory syndrome.

**Table 5:** Relationship between healthcare worker status and other prognostic variables

Variable	Non-HCW	HCW	p value
Age	42±14	37±11	0.007
Onset-to-treatment (d)	5.3±3.0	3.8±2.2	0.001
Neutrophil (x10^9^/L)	4.5±2.8	3.9±1.5	0.04
Lactate dehydrogenase (IU/L)	276±161	188±63	<0.001

^a^HCW, healthcare worker.

### Benefit of Treatment

Most of the patients in this cohort were treated according to protocol. The clinical outcome did not represent the natural history of SARS. The only variable that was related to the benefit of treatment was the time from onset to treatment. Donnelly et al. found that the time between the onset of symptoms and admission to hospital did not affect the death rate (*12*). In this study, patients who sought treatment early and received antiviral and steroid combination therapy were not shown to do better by multivariate analysis.

The Hospital Authority adopted an aggressive treatment protocol during the peak of the SARS epidemic in Hong Kong. Broad-spectrum antibiotics and a combination of ribavirin and steroid were the mainstays of treatment. The dose of ribavirin used was small to prevent major side effects. The administration of steroids in SARS treatment is controversial, however. Theoretically, the early use of steroids promotes viral replication, enhances infectivity, and possibly causes a rebound of infection. Peiris et al. found that the viral load peaked at day 10 in SARS patients treated with both ribavirin and steroids (*13*). However, immunosuppression or, more precisely, immunomodulation, is believed to be an effective therapy at the second stage of SARS. The current consensus among the Hospital Authority’s expert panel is to begin administering a steroid or pentaglobin at the second stage of SARS when a hypersensitivity immune reaction occurs (*8*).

Patients who sought treatment early tended to receive antiviral therapy at a later time. This is understandable since the symptoms of SARS are nonspecific, and clinicians also rely on laboratory data for diagnosis. The sensitivity of current RT-PCR assays is not satisfactory. A more sensitive and rapid diagnostic test must be developed, particularly if we have an effective treatment regime in the future.

## Conclusion

One third of the SARS patients in our study had a limited disease course. In the remaining two thirds, pneumonitis progressed rapidly after the early use of ribavirin and steroid combination therapy. Apparently, approximately one third responded to pulsed doses of steroids, while the other third depended on treatment with high amounts of oxygen. Intensive care was required for 21% of patients. Advanced age, high neutrophil count on admission, and elevated initial lactate dehydrogenase level were independent correlates of an adverse clinical outcome. Strong evidence to support early and routine use of ribavirin and steroid combination therapy in all SARS patients does not exist.

We need to investigate new antiviral agents and test the efficacy of steroids in randomized controlled trials. SARS is an entirely new emerging disease and its clinical course varies widely. By stratifying our patients according to risk, we could individualize our treatment protocol. In addition, we need a more sensitive and rapid diagnostic test for SARS-associated coronavirus infection, both for treatment and for forming cohorts of patients infected with this deadly disease.

## References

[1] Peiris JS, Lai ST, Poon LL, Guan Y, Yam LY, Lim W, Coronavirus as a possible cause of severe acute respiratory syndrome. Lancet. 2003;361:1319–25. 10.1016/S0140-6736(03)13077-2 12711465 PMC7112372

[2] Centers for Disease Control and Prevention. SARS coronavirus sequencing. [Accessed May 26, 2003] Available from: URL: http://www.cdc.gov/ncidod/sars/sequence.htm

[3] WHO. Interim guidelines for national SARS preparedness. [Accessed May 26, 2003] Available from: URL: http://www.wpro.who.int/sars/interim_guidelines/interim_guidelines_26May.pdf

[4] Lee N, Hui D, Wu A, Chan P, Cameron P, Joynt GM, A major outbreak of severe acute respiratory syndrome in Hong Kong. N Engl J Med. 2003;348:1986–94. 10.1056/NEJMoa030685 12682352

[5] Department of Health. HKSAR. Outbreak of severe acute respiratory syndrome (SARS) at Amoy Gardens, Kowloon Bay, Hong Kong. [Accessed June 2, 2003] Available from: URL: http://www.info.gov.hk/info/ap/pdf/amoy_e.pdf

[6] Hong Kong Hospital Authority. [Accessed May 28 2003] Available from: URLHong Kong Hospital Authority. Available from: URL: http://www.ha.org.hk

[7] Tsui PT, Mok NS, Kwan CP, Lee KK, Law CB, Lai ST. Wide spectrum of clinical course in subjects infected with SARS associated coronavirus: case report. In press

[8] Hong Kong Hospital Authority. HA information on management of SARS. [Accessed May 28, 2003] Available from: URL: http://ha.home/ho/ps/clinical_management-treatment.htm#principle

[9] Nicholls JM, Poon LLM, Lee KC, Ng WF, Lai ST, Leung CY, Lung pathology of fatal severe acute respiratory syndrome. Lancet. 2003;361:1773–8. 10.1016/S0140-6736(03)13413-7 12781536 PMC7112492

[10] World Health Organization. Severe acute respiratory syndrome (SARS): laboratory diagnostic tests. [Accessed June 2, 2003] Available from: URL: http://www.who.int/csr/sars/diagnostictests/en/

[11] Hong Kong Hospital Authority. HA information on management of SARS: infection control. [Accessed June 3, 2003] Available from: URL: http://ha.home/ho/ps/sars_infection_control.htm

[12] Donnelly CA, Ghani AC, Leung GM, Hedley AJ, Fraser C, Riley S, Epidemiological determinants of spread of causal agent of severe acute respiratory syndrome in Hong Kong. Lancet. 2003;361:1761–6. 10.1016/S0140-6736(03)13410-1 12781533 PMC7112380

[13] Peiris JS, Chu CM, Cheng VC, Chan KS, Hung IFN, Poon LLM, Clinical progression and viral load in a community outbreak of coronavirus-associated SARS pneumonia: a prospective study. Lancet. 2003;361:1767–72. 10.1016/S0140-6736(03)13412-5 12781535 PMC7112410

